# Enhanced serum interferon-lambda 1 interleukin-29 levels in patients with psoriasis vulgaris^☆^^☆☆^

**DOI:** 10.1016/j.abd.2020.11.007

**Published:** 2021-05-24

**Authors:** Li-xin Fu, Tao Chen, Zai-Pei Guo, Na Cao, Li-Wen Zhang, Pei-Mei Zhou

**Affiliations:** a Department of Dermatovenereology, Chengdu Second People’s Hospital, Chengdu, Sichuan, China; b Department of Dermatovenereology, West China Hospital of Sichuan University, Chengdu, Sichuan, China

**Keywords:** Interleukins, Leukocytes, mononuclear, Psoriasis

## Abstract

**Background:**

Interferon (IFN)-λ1, also named Interleukin (IL)-29, is a new member of the Type III IFN or IFN-λ family. IL-29 plays an important role in the pathogenesis of many types of autoimmune and inflammatory diseases.

**Objective:**

To study the role of IL-29 in the pathogenesis of psoriasis vulgaris.

**Methods:**

The authors detected the serum levels of IL-29 in forty-one patients with psoriasis vulgaris, twenty-three patients with atopic dermatitis and thirty-eight age and gender-matched controls by sandwich Enzyme-Linked Immunosorbent Assay (ELISA). The effects of IL-29 on the expression of cytokines, such as IL-6, IL-17, IL-8, IL-4, IL10, Interferon (IFN-γ) and Tumor Necrosis Factor-α (TNF-α), in PBMCs and HaCat cells were determined by real-time quantitative PCR.

**Results:**

Our data indicated that serum IL-29 levels were significantly elevated in patients with psoriasis vulgaris when compared with atopic dermatitis patients and the control group. Moreover, Serum levels of IL-29 were closely associated with the severity of psoriasis vulgaris. Furthermore, IL-29 up-regulated the mRNA expression levels of IL-6, IL-17 and TNF-α in PBMCs from psoriasis vulgaris patients. In addition, IL-29 enhanced the IL-6 and IL-8 expression from the HaCat cells.

**Conclusion:**

This study provides the first observations on the association of IL-29 and psoriasis vulgaris and showed elevated IL-29 serum levels. The authors suggest that IL-29 may play a role in the pathogenesis of psoriasis vulgaris.

## Introduction

Psoriasis is a common T-cell-mediated chronic inflammatory skin disorder, histopathologically characterized by hyper-proliferation of keratinocytes and dermal vascular, infiltration with mononuclear cells.^1^, ^2^, ^3^, ^4^ However, the molecular mechanisms in the development of this disease have not been entirely elucidated.

Interferon (IFN)-λ1, also named Interleukin (IL)-29, is a new member of Type III IFN or IFN-λ family. The family of Type III IFN is comprised IFN-λ1 (IL-29), IFN-λ2 (IL-28A) and IFN-λ3 (IL-28B). Many investigators have demonstrated that IL-29 plays an important role in the pathogenesis of many types of autoimmune and inflammatory diseases, such as systemic sclerosis, rheumatoid arthritis and systemic lupus erythematosus.^5^, ^6^, ^7^ However, the association of IL-29 with Psoriasis Vulgaris (PV) is still poorly understood. Thus, in this study, the authors compared the IL-29 serum levels in PV patients with healthy controls.

More recently, accumulating research has indicated that IL-29 can affect the cytokines production by activating the Janus Kinase/signal transducer and activators of the transcription pathway. IL-29 upregulated IL-1β, IL-8, MCP-1 expression and promoted monocyte/macrophage migration in human Simpson-Golabi-Behmel syndrome.^8^ IL-29 can increase the production of IL-10, IL-8 and IL-6 in macrophages and inhibits the release of IL-13 in T-cells.^9^, ^10^, ^11^ Moreover, He et al reported that IL-29 could induce the secretion of IL-6 and TNF from CD14+T-cells as well as IL-4 and IL-13 from mast cells.^12^ Keratinocytes, as a major cell of innate immunity, play an important role in skin inflammation and immune response by expressing cytokines and antimicrobial peptides in psoriasis. HaCat cells are one type of keratinocyte cell line. To study the role of IL-29 in the pathogenesis of PV, the authors investigate the effects of IL-29 on mRNA expression of cytokines (including IL-6, TNF-α, IL-17, IL-8, IL-4, IFN-γ, and IL10) in PBMCs from PV patients and keratinocyte cell line HaCaT in this study.

## Methods

### Patients

Forty-one patients with PV, twenty-three patients with Atopic Dermatitis (AD), and thirty-eight age and gender-matched controls were enrolled in this study. The detailed history for each patient was provided in Table 1. The severity of disease in patients with PV or AD was determined using Psoriasis Area and Severity Index (PASI) or scoring AD (SCORAD). Among them, 17 PV patients were treated with topical steroid and tar, and oral compound glycyrrhizin (Each table contains Glycymhizin 25 mg, Monoammonium Glycyrrhizinate 35 mg, Glycine 25 mg, and DL-Methionine 25 mg. The patients were treated with two tables one time, triple daily. Nippon Kayaku, Tokyo, Japan). The serum samples of these patients were collected both before and after treatment. In addition, heparinized blood samples were collected from 16 PV patients. There was not any history of recent viral infections or lymphocytosis or lymphopenia or any other correlating investigation in these patients. All the PV patients were strictly psoriasis vulgaris only, without any other phenotypes overlap.

**Table 1:** Cohort demographics.

	PV	AD	Controls
Number	41	23	38
Sex	21 M / 20 F	11 M / 12 F	20 M / 18 F
Disease activity	15.71 (4.8–36.4)^a^	34.87 (18.5–67)^b^	NA

PV, Psoriasis Vulgaris; AD, Atopic Dermatitis; NA, Not Applicable.

a Disease activity in PV patients was assessed by the Psoriasis Area and Severity Index (PASI).

b Disease activity in AD patients was assessed by scoring AD (SCORAD) index.

### Assay for serum IL-29 levels

Serum IL-29 levels in a different group of patients were measured by commercially available Enzyme-Linked Immunosorbent Assay (ELISA) kits (Boster, Wuhan, China) following the manufacturer’s instruction.

### Cell culture

PBMCs of PV patients were isolated from collected peripheral blood by gradient density centrifugation using commercially available kits (Tianjin Haoyang Biological Manufacture Co. Ltd., Tianjin, China). The isolated PBMCs from each patient were divided into two groups and cultured in DMEM containing 10% FBS, 100 mg/mL penicillin and streptomycin in a 6-well plate at 1 mL per well. One group of cells was treated with recombinant 100 ng/mL IL-29 (Peprotech, London, UK) for 6 h, and the other group was treated with PBS as control. After treatment, total RNA was extracted by Trizol reagent (Invitrogen Corp, Carlsbad, CA, USA) according to the manufacturer's instructions.

HaCaT cells were cultured in DMEM containing 10% FBS, 100 mg/mL penicillin and streptomycin and treated with recombinant IL-29 (1 ng/mL, 10 ng/mL, 100 ng/mL, 1000 ng/mL) or TNF-α (10 ng/mL) for 2 h. Cells were treated with PBS as control. After treatment, the total RNA of cells was extracted by Trizol reagent according to the manufacturer's instructions.

### Real-time quantitative PCR

The mRNA levels of IL-6, TNF-α, IL-17, IL-4, IFN-γ, IL-10, and GAPDH in PBMCs after incubated with IL-29 or PBS and the expression of IL-6, IL-8 and GAPDH in HaCat cells after incubated with IL-29, TNF-α or PBS was determined by real-time quantitative PCR. The cDNA was synthesized from the total RNA by using RT reagent Kit with gDNA Eraser (Takara, Dalian, China). cDNA samples were amplified in a 20 μL reaction volume containing 10 μL of 2 × SYBR Green Master Mix (Takara, Dalian, China), 2 μL of cDNA and 0.25 μM qPCR primers. The following primers were used: IL-4 (P216616, Bioneer, Inc., Daejeon, Korea); IL-6 (P211161, Bioneer, Inc., Daejeon, Korea); IL-10 (P285360, Bioneer, Inc., Daejeon, Korea); IL-17 (P291322, Bioneer, Inc., Daejeon, Korea); IFN-γ (Catalog: HQP009467, GeneCopoeia, USA); TNF-α (P237423, Bioneer, Inc., Daejeon, Korea); GAPDH (5’-CGGAGTCAACGGATTTGGTC-3’ and 5’-CGGTGCCATGGAATTTGCCA-3’). The conditions were 95 °C for 5 min, and 95 °C for 15 s and 60 °C for the 30 s of 40 cycles with a final extension at 72 °C for 5 min. The mRNA levels of IL-6, TNF-α, IL-17, IL-4, IFN-γ, IL-10, and IL-8 were expressed as relative mRNA levels compared with control and determined by the 2-ΔΔCt method.

### Statistical analysis

Each experiment was carried out at least three times. The data were expressed as mean ± SD, Statistical differences between groups were determined according to one-way analysis of variance, *t*-test or Mann-Whitney *U* Test. Paired data were compared by Wilcoxon signed-rank test. The association of IL-29 with PASI scores was analyzed by the Spearman test; p < 0.05 was set as statistically significant.

## Results

### Elevated IL-29 serum levels in PV patients

**Figure 1:**
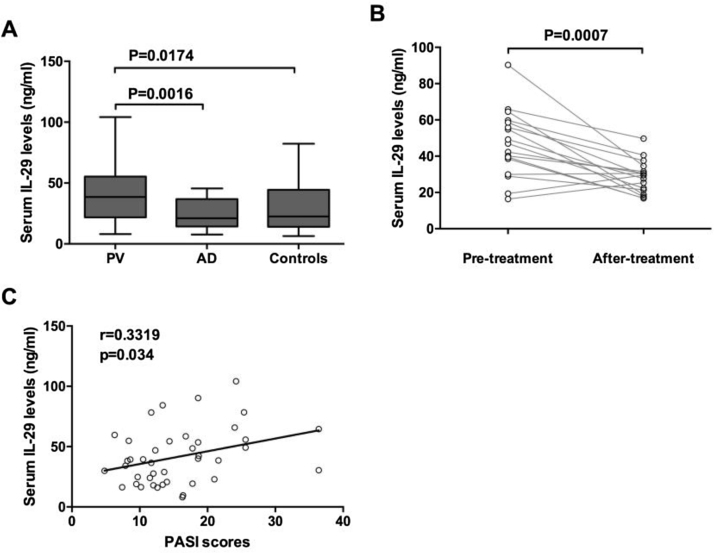
Increased serum IL-29 levels in PV patients. Serum levels of IL-29 in patients with PV and AD and healthy controls were determined by ELISA. (A) Serum IL-29 levels in patients with PV in the acute stage were significantly higher than those in AD patients and control subjects; p-values are based on the Mann-Whitney *U* Test. (B) Wilcoxon Signed Ranks Test for paired data showed that serum IL-29 levels of 17 patients with PV in the acute stage were significantly higher than those in the convalescent stage. (C) A positive correlation was shown between IL-29 serum levels and PASI scores in PV patients by Spearman tests.

The serum levels of IL-29 in patients with PV and AD and control were detected by ELISA. As shown in Fig. 1A, IL-29 serum levels in patients with PV (41.62 ± 23.26 ng/mL) were significantly higher than those in AD patients (24.35 ± 12.57 ng/mL) and control group (29.83 ± 19.52 ng/mL). Moreover, 17 PV patients had obviously elevated serum IL-29 levels when compared with those after treatment (Fig. 1B); (p = 0.0007). Furthermore, as presented in Fig. 1C, serum IL-29 levels in PV patients were correlated with PASI scores (*r* = 0.3319, p = 0.034).

### IL-29 up-regulates the mRNA expression of IL-6, TNF-α, and IL-17 in PBMCs from PV patients

**Figure 2:**
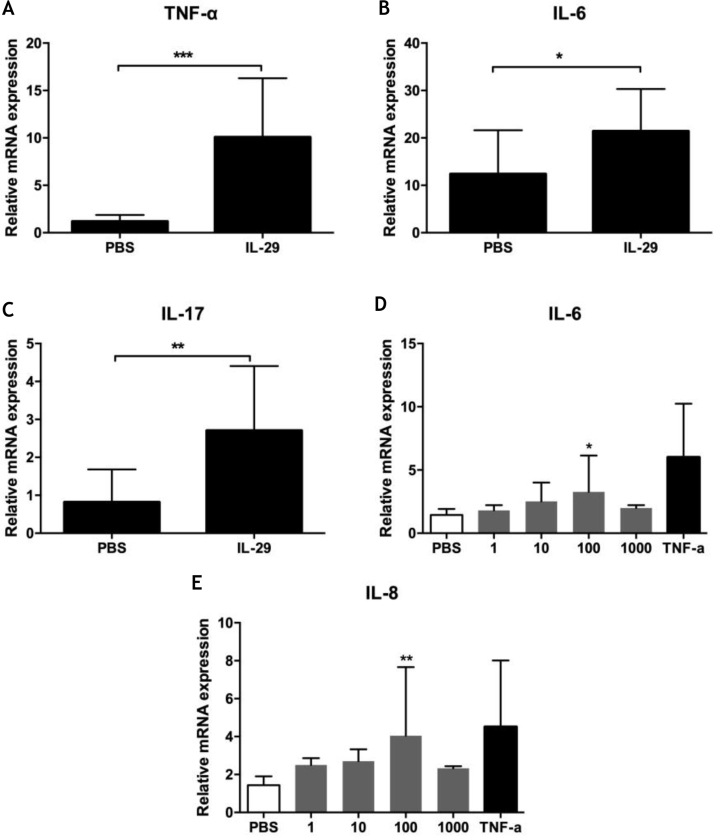
(A–C) IL-29 induced IL-6, IL-17 and TNF-α mRNA expression in PBMCs from PV patients. PBMCs obtained from 10 PV patients were treated with IL-29 100 ng/mL or PBS for 6 h. After that, mRNA expression of IL-6, IL-17 and TNF-α in PBMCs were measured by Real-time quantitative PCR. IL-29 obviously enhanced IL-6, IL-17 and TNF-α mRNA expression in PBMCs; p-values are based on *t-*test. n = 10, *p < 0.05, **p < 0.01, compared with control group. (D–E) IL-29 enhanced IL-6 and IL-8 mRNA expression in HaCat cells. HaCaT cells were treated with recombinant IL-29 (1 ng/mL, 10 ng/mL, 100 ng/mL, 1000 ng/mL) or TNF-α (10 ng/mL) for 2 h. After that, mRNA expression of IL-6 and IL-8 in HaCat cells was measured by Real-time quantitative PCR. IL-29 obviously enhanced IL-6 and IL-8 mRNA in HaCat cells; p-values are based on the One-way analysis of variance. n = 10, *p < 0.05, **p < 0.01, compared with control group. All data are expressed as mean ± SD.

To explore the role of IL-29 in the pathogenesis of PV, the authors assayed the effects of IL-29 on cytokines mRNA expression (including IL-6, TNF-α, IL-17, IL-4, IFN-γ, and IL-10) in PBMCs from 10 PV patients by real-time quantitative PCR. As presented in Fig. 2A–C, IL-29 caused a markedly increase in the mRNA levels of IL-6, TNF-α and IL-17 in PBMCs from PV patients treated with IL-29 when compared with PBS controls. However, mRNA expression of IL-4, IL10, and IFN-γ were no changes in PBMCs from PV patients after IL-29 incubated (data not shown).

### IL-29 enhances IL-6 and IL-8 expression in HaCat cells

To explore the function of IL-29 in the pathogenesis of PV, the authors also assayed cytokines mRNA expression (IL-6 and IL-8) in Hacat cells after treatment with recombinant IL-29 by real-time quantitative PCR. As presented in Figs. 2D and 2E, IL-29 caused an obvious increase in the mRNA levels of IL-6 and IL-8 in HaCat cells treated with IL-29 when compared with PBS controls.

## Discussion

As described above, IL-29, a new member of the type III IFN cytokine family, is closely related to the pathogenesis of many types of autoimmune and inflammatory diseases.^5^, ^7^, ^13^, ^14^ In this study, the increased serum levels of IL-29 were founded in PV patients when compared with those in AD patients and healthy controls. Moreover, IL-29 serum levels were correlated with the severity of PV. Data suggest that IL-29 may play a role in the pathogenesis of PV.

Many researchers reported that IL-29 can be expressed in many cell types and be displayed immunoregulatory activities in naïve PBMCs.^1^, ^15^ Wang et al. have demonstrated that IL-29 enhances TLR-induced proinflammatory cytokine production in RA-FLS via up-regulation of TLRs.^16^ Gallagher et al. have reported that IL-29 appears to be an inhibitor of human Th2 responses whose action is primarily directed towards IL-13 but which may also affect Th2 responses generally and does not invoke a complementary elevation of IFN-γ secretion.^10^ Yan et al. have shown that IL-29 combined with rhGM-CSF and IL-4 can induce DC maturation, increase the expression of the costimulatory molecules and stimulate cytokine and chemokine production in the immune tolerant phase and the immune clearance phase.^17^ Lots of studies had shown that Th1/Th17 cells played crucial roles in the pathogenesis of psoriasis.^18^, ^19^, ^20^ Psoriatic lesions can secrete many inflammatory cytokines such as IFN-γ, IL-6, IL-1 β, IL-8, IL-17, and TNF-α, which contribute to the development of psoriasis.^21^ In this study, the authors investigated the effects of IL-29 on mRNA expression of cytokines (including IL-6, TNF-α, IL-17, IL-4, IFN-γ, and IL-10) in PBMCs from PV patients and HaCat cells treated with IL-29. The authors indicated that IL-29 markedly enhanced the mRNA expression of TNF-α, IL-6 and IL-17 in PBMCs from PV patients. The authors also found that IL-29 obviously increased the mRNA expression of IL-6 and IL-8 in HaCat cells. A previous study indicated that TNF-α, IL-6, IL-8, and IL-17 play a crucial role in the pathogenesis of PV.^18^, ^22^ Hence, the authors suggest that IL-29 may up-regulate the expression of cytokines in PBMCs and keratinocytes, and enlarge inflammatory reaction in the pathogenesis of PV.

## Conclusion

Taken together, this study provides first observations on the association of IL-29 and PV and showed the elevated IL-29 serum levels in PV patients. The authors of this present study suggest that IL-29 may serve as a pro-inflammatory cytokine and play a role in the pathogenesis of PV.

## Ethics statement

Human research was approved by the Ethics Committee of Chengdu Second People’s Hospital (nº 2015007). All patients were oriental and gave written informed consent.

## Financial support

The study was supported, in part, by the 10.13039/501100001809Natural Science Foundation of China (81470143), and the 10.13039/501100004829Sichuan Provincial Department of Science and Technology (2019YJ0627).

## Authors’ contributions

Li-xin Fu: Concept, design, definition of intellectual content; literature search; experiment studies; manuscript preparation; manuscript editing.

Tao Chen: Concept, design, definition of intellectual content; manuscript preparation; manuscript editing.

Zai-pei Guo: Concept, design, definition of intellectual content; manuscript review.

Na Cao: Data acquisition and data analysis.

Li-wen Zhang: Literature search; Experiment studies.

Pei-mei Zhou: Data acquisition and data analysis.

## Conflict of interest

None declared.
